# Extending Catalyst Life in Glycerol-to-Acrolein Conversion Using Non-thermal Plasma

**DOI:** 10.3389/fchem.2019.00108

**Published:** 2019-03-01

**Authors:** Lu Liu, Xiaofei Philip Ye, Benjamin Katryniok, Mickaël Capron, Sébastien Paul, Franck Dumeignil

**Affiliations:** ^1^Department of Biosystems Engineering and Soil Science, The University of Tennessee, Knoxville, TN, United States; ^2^Univ. Lille, CNRS, Centrale Lille, ENSCL, Univ. Artois, UMR 8181 - UCCS - Unité de Catalyse et Chimie du Solide, Lille, France

**Keywords:** non-thermal plasma, glycerol, acrolein, coking, deactivation, catalyst regeneration

## Abstract

Booming biodiesel production worldwide demands valorization of its byproduct of glycerol. Acrolein, an important intermediate chemical, can be produced by gas-phase glycerol dehydration catalyzed by solid acids. Because catalysts that lead to high acrolein selectivity usually deactivate rapidly due to the formation of coke that blocks the active sites on their surface, one major challenge of this method is how to extend the service life of the catalyst. Silica-supported silicotungstic acid (HSiW-Si) is a good example of such a catalyst that shows good activity in glycerol dehydration to acrolein initially, but deactivates quickly. In this study, HSiW-Si was selected to probe the potential of using non-thermal plasma with oxygen-containing gas as the discharge gas (NTP-O_2_) to solve the catalyst deactivation problem. NTP-O_2_ was found to be effective in coke removal and catalyst regeneration at low temperatures without damaging the Keggin structure of the HSiW-Si catalyst.

## Introduction

Acrolein, the simplest unsaturated aldehyde, is an important intermediate leading to many useful chemicals and materials, such as methionine, and acrylic acid and its esters (Liu et al., 2012). The manufacturing method for acrolein has been partial oxidation of propylene since the 1950s (Weigert and Haschke, 1976). The market demand for propylene far exceeds its availability. Securing this raw material supply is now becoming highly strategic in a tough competition among downstream applications. Recently, an alternative scheme for acrolein production from glycerol has become a promising candidate, since glycerol, which is co-produced with biodiesel, has become abundantly available (Cheng et al., 2013; Katryniok et al., 2013; Zou et al., 2016). This value-added synthesis from glycerol to acrolein could alleviate propylene supply issue while providing a sustainable approach for backing up the biodiesel industry viability.

Highly efficient solid acid catalysts are the key in the gas-phase reaction to produce bio-based acrolein from glycerol. Suitable acidity of the catalysts leads to a high selectivity to acrolein, but unfortunately also results in severe coke formation, and consequently, the deactivation of the catalysts (Alhanash et al., 2010). Good glycerol conversion and acrolein yield have been achieved with many solid acid catalysts (Chai et al., 2007; Tsukuda et al., 2007; Atia et al., 2008; Alhanash et al., 2010), with the major remaining challenge being how to extend the service life of the catalyst (Katryniok et al., 2009, 2010, 2013; Liu et al., 2012). If the catalyst deactivation problem cannot be solved, industrialization of this bio-based acrolein production from glycerol is not promising. Therefore, studies focusing on extending the catalyst life are necessary.

Silicotungstic acid (H_4_SiW_12_O_40_, hereafter abbreviated as HSiW) is a commercially available heteropoly acid, and supported HSiW has been proven as one of the best performers among many studied catalysts in achieving high yield for the glycerol-to-acrolein conversion (Tsukuda et al., 2007; Atia et al., 2008; Liu et al., 2012). However, because of its strong acidity, supported HSiW unavoidably suffers from severe deactivation due to coke formation. The Keggin structure (Wu et al., 1996; Timofeeva, 2003), the most important feature of HSiW, is retained intact after coke formation (Kozhevnikov, 2007). Deposited coke has been reported to occur as an amorphous form, and its existence would not alter any crystalline structure of the catalyst (Wang et al., 2009, 2010; Alhanash et al., 2010). Therefore, supported HSiW can be regenerated to regain the acid catalytic activity if the deposited coke is removed.

Carbonaceous coke is a distribution of polynuclear aromatic substances of aliphatic and alicyclic compounds usually formed via condensation, hydrogen abstraction, polymerization, and repetitions of these reactions (Khan and Al-Jalal, 2008). The terms “soft” and “hard” refer to the relative properties of coke those are associated with the structural complexity and degree of polymerization of the carbonaceous substances. “Harder” coke is more complex in structure and more difficult to burn off. Catalyst deactivation due to coking is common in industrial chemical production processes; an example is catalytic cracking of petroleum fractions (Khan and Al-Jalal, 2008). The routine treatment is to flush the catalyst bed with oxygen-containing gas at elevated temperatures, usually 450–600°C, but depending on the catalyst characteristics and specific reactions, higher temperatures (600–800°C) may be required to remove the coke (Tanabe, 1989; Khan and Al-Jalal, 2004; Chai et al., 2008). A problem encountered is that the Keggin structure of HSiW can only be maintained up to 400°C (Atia et al., 2008) [one study claims up to only 300°C (Bardin and Davis, 2000)], depending on the characteristics of the supports. Consequently, regeneration using the high-temperature combustion approach will remove the coke only at the cost of reducing catalytic activity afterwards, due to the damage of the Keggin structure and consequently significant loss of activity (Atia et al., 2008; Katryniok et al., 2012). Therefore, this conventional decoking treatment is not suitable for regenerating supported HSiW for the acrolein production process.

Non-thermal plasma (NTP) can initiate the formation of ions, free radicals, and other highly reactive intermediates at low temperatures. In an O_2_-containing atmosphere, NTP can convert the O_2_ molecule into many highly reactive oxygen species, including the triplet ground-state oxygen atom O(^3^P), the metastable oxygen atom O(^1^D), the metastable oxygen molecule O_2_(^1^Δg), and ozone O_3_ (Fridman, 2008). When humidity is present, additional highly reactive species are formed, such as H_2_O^+^, free H atoms, and OH radicals (Fridman, 2008). Most of these species are highly oxidative and can oxidize substances that fail to be oxidized by O_2_ or air under mild temperature conditions. Therefore, non-thermal plasma with oxygen-containing gas as the discharge gas (NTP-O_2_) is very likely to contribute to solving the deactivation problem of a solid acid catalyst having low thermal stability. Regenerating deactivated catalysts is a challenging issue of industrial importance; depending on the catalyst's characteristics and the reaction in which it is used, the deactivated catalysts can vary significantly in their coke profile, and thus they may vary in term of how difficult it is for them to be regenerated (Silva et al., 2004). Therefore, it is worth expending some effort on regenerating a promising acid catalyst deactivated in the glycerol-dehydration process.

The objective of this study was to investigate the effectiveness of NTP-O_2_ in the coke removal (decoking) and regeneration of deactivated solid acid catalysts to increase the potential of bio-based acrolein production in industrial applications.

## Materials and Methods

### Catalyst and Catalytic Reaction

Silica-supported silicotungstic acid (HSiW-Si) would be a good catalyst for acrolein production from glycerol if it did not deactivate so quickly (Tsukuda et al., 2007). Therefore, HSiW-Si was chosen as an excellent candidate to explore the potential of applying NTP-O_2_ to extending catalyst service life. The experiments in this study were designed to evaluate whether NTP-O_2_ can be effective in removing the coke from catalyst surface and regenerating the deactivated catalyst. Ultimately, the method developed in this study for glycerol dehydration to acrolein could be applied to other catalytic reactions using solid catalysts that have relatively low thermal stability.

Mesoporous silica granules (SiO_2_, Davicat® Si1252) were supplied by Grace-Davison (Columbia, MD, USA). The silica granules have particle sizes of 1 to 3 mm, an average pore diameter of 11 nm, and an average surface area of 390 m^2^/g. Silicotungstic acid (HSiW) was purchased from Sigma Aldrich (St. Louis, MO, USA), and it was loaded on the catalyst support by the impregnation method. Briefly, HSiW of 10 wt.% on the basis of the silica support (Si) was dissolved in deionized water to make a 0.04 g/mL solution. The silica, which was previously calcined at 300°C in a muffle furnace for 2 h, was added to the HSiW solution. The mixture was set at room temperature for 24 h to reach the adsorption-desorption equilibrium. The resultant HSiW-Si solids were first dried at 55°C for 24 h, followed by further drying at 105°C for 6 h to complete dryness, and then calcined at 300°C in a muffle furnace for 2 h before the 2nd impregnation. The procedures were repeated for another 10 wt.% HSiW loading. Hence, a catalyst consisting of 20 wt.% HSiW on silica was obtained.

**Figure 1:**
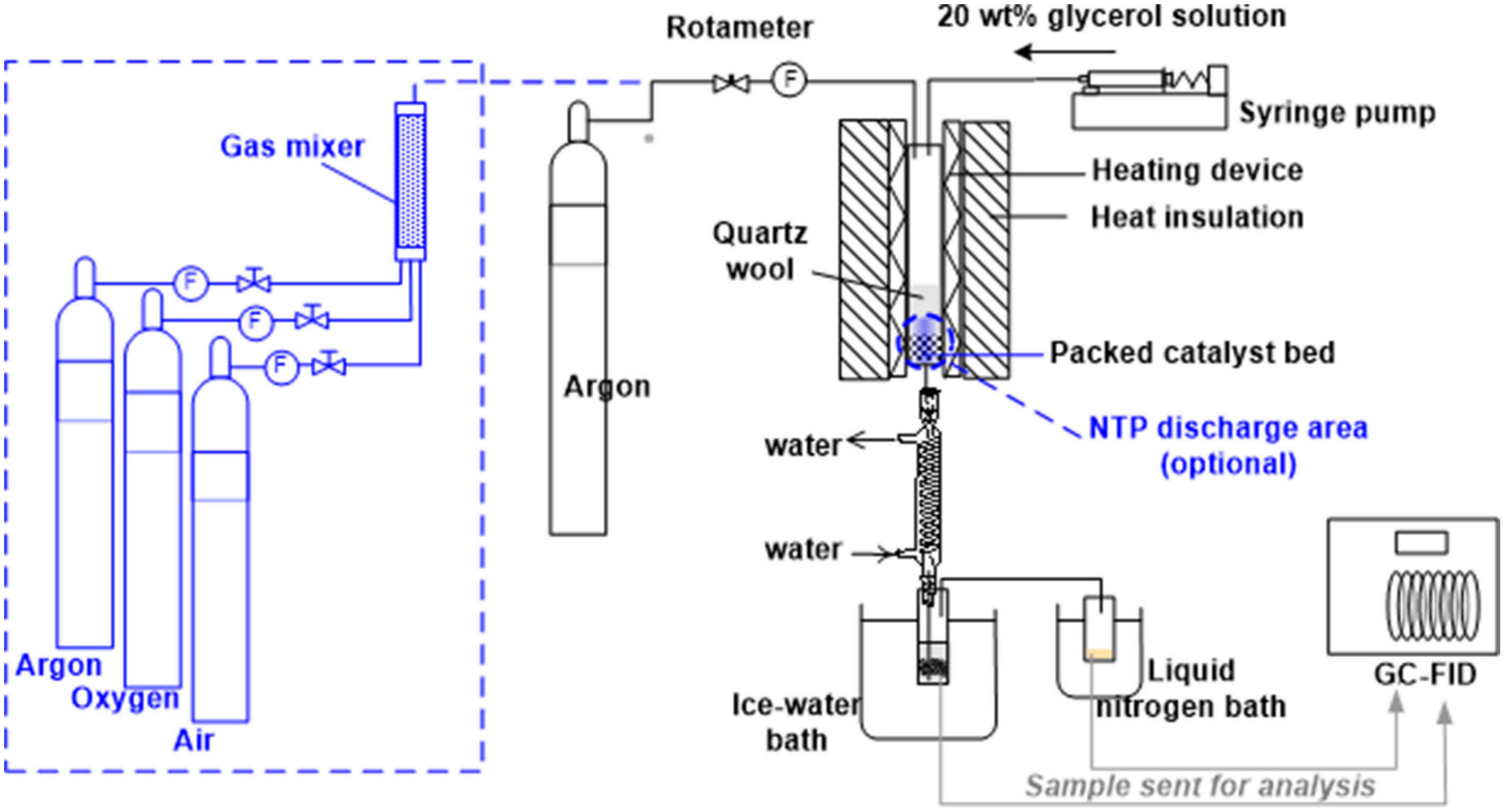
Experimental setup for glycerol dehydration at reaction temperature of 275°C (The optional elements inside dashed lines were used for the experiments described in section Evaluation of *in situ* Catalyst Regeneration by NTP).

The as-obtained silica-supported HSiW catalyst (HSiW-Si) was used as a catalyst for the glycerol dehydration reaction (275°C, 7.5 h Time-On-Stream) that was carried out in a down-flow packed-bed reactor at atmospheric pressure (Figure 1). The reactor was made of a quartz tube (length 300 mm, ID 19.35 mm, OD 25.3 mm), which was heated by a heating tape evenly wrapped around the outer wall of the quartz tube. The heating tape was controlled by a PID temperature controller to maintain the desired temperature of the catalyst bed. The reactor had an external layer of thermal insulation to minimize the heat loss to the surroundings. Seven milliliter of HSiW-Si (~3.2 g) were packed at the lower end of the reactor, leaving a sufficient path length for the carrier gas and the glycerol feed to be preheated to the desired temperature before reaching the catalyst bed. The glycerol solution (20 wt.% of glycerol in water) was fed by a syringe pump at 6 mL/h feeding rate, resulting in an 84 h^−1^ gas hourly space velocity (GHSV) of glycerol. The flow rate of carrier gas argon (Ar) was regulated at 60 mL/min. The temperature of the catalyst bed was controlled at 275°C during the glycerol dehydration. Reaction effluent was first passed through a condenser with flowing tap water, and the majority of the liquid product was condensed in a 50 mL vial immersed in an ice-water cold bath (1st condensation stage). Any product that was not condensed in the first condensation stage was collected in a 20 mL vial that was immersed in a liquid nitrogen bath (2nd condensation stage). During the dehydration reaction, samples were collected every 1.5 h and analyzed using a gas chromatography equipped with a flame ionization detector (GC-FID) and a VB-WAX capillary column (Valco Instrument Co. Inc., USA).

Powder X-ray diffraction (XRD) was used to examine whether the silica-supported HSiW underwent any structural change after being used in the dehydration reaction. Solid-State Cross-Polarization Magic Angle Spinning Carbon-13 Nuclear Magnetic Resonance (CP/MAS ^13^C-NMR) was performed on spent HSiW-Si with and without NTP-O_2_ treatment to study the change of coke structure (Supplementary Material).

### NTP Generation

**Figure 2:**
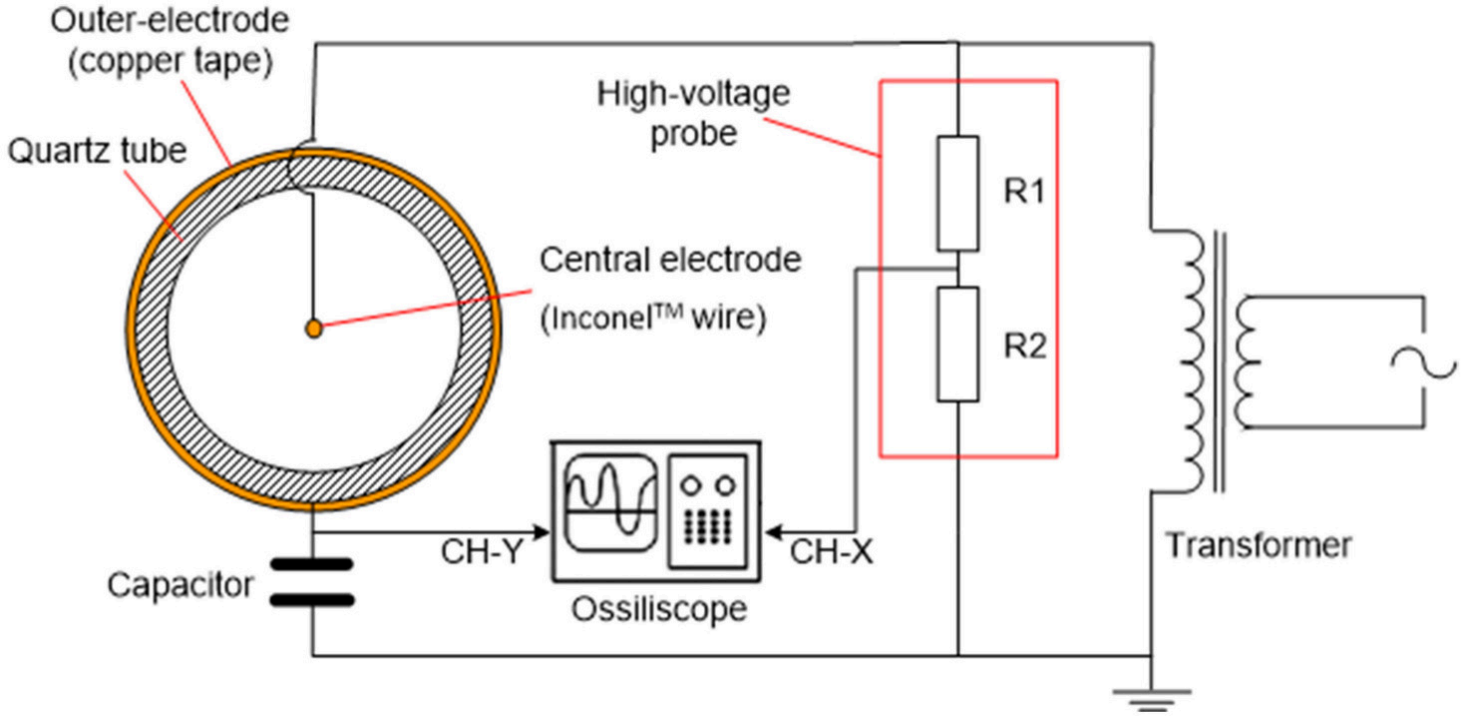
Schematics of the electrical setup for the NTP generation and the section of DBD cell.

Non-thermal plasma was generated by a wire-to-cylinder configuration of dielectric barrier discharge (DBD) as shown in Figure 2. DBD is a simple NTP generation approach that can be found in many other applications [e.g., (Kogelschat, 2002)]. An Inconel wire was inserted in the axial direction of the quartz reactor tube, and connected to the high-voltage port of a transformer. Copper tape was wrapped around the outside wall of the quartz tube, and connected to the ground. The wire and copper tape served as two electrodes, while the quartz wall served as the dielectric barrier. AC waveform was generated by an inverter (solid-state drive SSD110 model, PTI, Racine, WI) followed by a voltage transformer (55-HLH10102/D115, PTI, Racine, WI). The waveform of the NTP discharge was monitored via an oscilloscope (WaveLet™ series, LeCroy, Chestnut Ridge, NY). A capacitor (2 μF) was inserted in the circuit to create a phase lag to generate Lissajous figure for the calculation of power consumption by NTP (Manley, 1943; Kraus et al., 2001; Subrahmanyam et al., 2005).

### Evaluation of Coke Removal by NTP

We first designed two types of tests to evaluate NTP's effect in coke removal.

**Figure 3:**
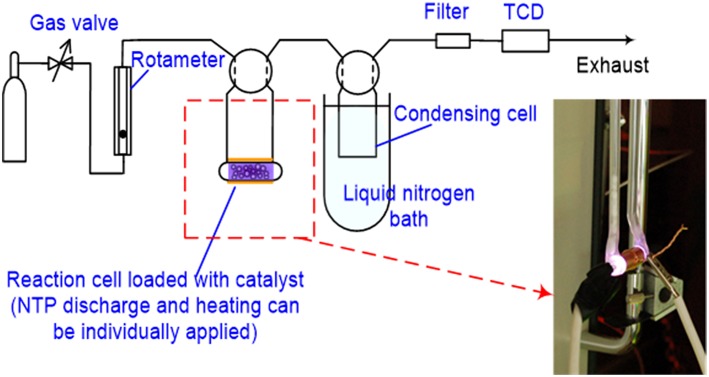
Schematics of the experiment setup to monitor NTP decoking process using TPR.

Test 1: Monitoring NTP decoking process using temperature programmed reaction (TPR)
    This test was completed using a system that was constructed based on a ChemBET PULSAR™ TPR/TPD (Quantachrome Instruments, Boynton Beach, FL, USA) with a homemade DBD cell (electrical configuration is depicted in Figure 2). Schematic flow diagram of the test is shown in Figure 3. The spent HSiW-Si was collected from a glycerol dehydration run after 7.5 h Time-On-Stream (TOS) as described in section Catalyst and Catalytic Reaction; 100 mg of this spent catalyst was placed in the DBD cell, and heat could be applied as an option using a heating tape. A gas blend (5% O_2_ in He) was fed into the system at 30 mL/min, going through the DBD cell, and traveling through a liquid nitrogen (LN) trap before reaching a thermal conductivity detector (TCD). Helium could not be condensed in the LN trap and would pass through. Oxygen could also pass through the LN trap, because the 5% concentration was far below its saturation vapor pressure (Bayraktar and Kugler, 2002). Four NTP discharge field strengths (3.0, 4.8, 6.6, and 8.4 kV/cm), which were defined as the discharge voltage divided by the discharge gap length, were first studied at ambient temperature (25°C). Then the optimal NTP field strength was used to study the temperature effect at 25, 100, 150, and 200°C. A blank test was first conducted using fresh catalyst that was not used in a dehydration reaction. The DBD cell was loaded with 100 mg fresh catalyst with only helium or a 5% O_2_ in He gas blend flowing through the cell; NTP discharge was applied to the cell at the designed field strength levels. The purpose of the blank test was to obtain the baseline signal for each discharge strength. Thus, the TCD signal was able to qualitatively reflect the decoking process when a spent catalyst is used.
Test 2: Evaluating NTP de-coked spent catalyst using temperature programmed oxidation (TPO).
    The amount of coke removal by NTP-O_2_ was also evaluated by temperature-programmed oxidation (TPO), characterizing the spent catalyst after a specific NTP decoking treatment. Characteristics of the remaining coke on the catalyst surface after NTP-O_2_ treatment were obtained to evaluate the coke-removal effectiveness of each NTP-O_2_ condition (from Test 1).
    The experiment was conducted with the ChemBET PULSAR™ TPR/TPD, and the flow diagram is similar to Figure 3; the only difference was that instead of the NTP application, programmed heating was applied to the reaction cell. Catalyst sample (100 mg) was pretreated on the ChemBET at 300°C in nitrogen atmosphere prior to the TPO experiment, which was conducted with a temperature program from 25 to 900°C at a heating rate of 10°C/min. A gas blend (5% O_2_ in He) was fed into the reaction cell at 70 mL/min; the effluent gas was then passing through a liquid nitrogen trap before reaching the TCD detector. Oxidation products such CO_x_ was condensed, but the background gas helium and the unconsumed oxygen could pass through and detected by the TCD.

### Evaluation of *in situ* Catalyst Regeneration by NTP

This part of experiments focused on the catalyst performance for glycerol dehydration after *in situ* NTP regeneration treatment. The catalyst was kept inside the packed-bed reactor (Figure 1) and regeneration with NTP-O_2_ treatment was directly applied *in situ*. This was an indirect evaluation in term of coke removal, but a direct evaluation in term of whether the catalyst could be regenerated and the catalyst's performance could be regained.

The experiment proceeded as follows: after 7.5 h of glycerol dehydration as described in section Catalyst and Catalytic Reaction, the glycerol feed was stopped. Instead of pure argon, different oxygen-containing gases were fed into the reactor, and NTP discharge was applied to the catalyst bed region with the aim of regenerating the catalyst. After 2 h of the NTP-O_2_ application, NTP was stopped; the carrier gas was changed back to pure argon, and the glycerol feed was restarted. The glycerol-dehydration reaction was continued for a few hours, and glycerol conversion (Equation 1) and acrolein selectivity (Equation 2) were used to evaluate the catalyst performance.

(1) $$X_{glycerol}=\frac{n_{reacted}}{n_{feed}}\times 100\%=\frac{n_{feed}-n_{quantified}}{n_{feed}}\times 100\%$$

where *X*_*glycerol*_ is glycerol conversion (mol%), *n*_*reacted*_ is the moles of glycerol reacted, and *n*_*feed*_ is the moles of glycerol in the feed, *n*_*quantified*_ is the remaining glycerol in the collected sample quantified by GC.

(2) $$S_{acrolein}=\frac{n_{c-acrolein}}{n_{c-gly-reacted}}\times 100\%$$

where *S*_*acrolein*_ is the selectivity to acrolein (mol%), *n*_*c*−*acrolein*_ is the moles of carbon in the produced acrolein, and *n*_*c*−*gly*−*reacted*_ is the moles of carbon in the converted glycerol.

The elements inside dash lines in Figure 1 are designed for the experiment described in this section. NTP was integrated into the reactor system using the electrical setup as described in Figure 2, and the discharge zone started from 25.4 mm upstream of the catalyst bed and terminated at the end of the catalyst bed (a cylindrical NTP zone with 19.35 mm in diameter and 50.8 mm in height), including both a pre-bed discharge and an on-bed discharge zone.

The selected NTP field strength and operating temperature were based on the optimal condition found after evaluating the coke removal (section Evaluation of Coke Removal by NTP). Three different oxygen-containing discharge gases were compared: (1) 20% oxygen blended in argon, (2) air, which can be viewed as ~20% oxygen blended in nitrogen, and (3) pure oxygen. The total gas flow rate was fixed at 30 mL/min.

## Results

### Overview

The X-ray diffraction results (Supplementary Figure S1) showed no difference between the fresh HSiW-Si and the spent HSiW-Si that was collected after a 7.5-h glycerol dehydration test (section Catalyst and Catalytic Reaction); this result agreed with previous publications (Wang et al., 2009, 2010; Alhanash et al., 2010) in that the deposited coke was amorphous and would not change the crystalline structure of the catalyst on which it was deposited.

CP/MAS ^13^C-NMR analysis revealed that the relative abundance of aromatic and unsaturated C in NTP-treated spent catalyst decreased while that of C connected to heteroatoms of O significantly increased (Supplementary Figure S2), comparing with spent catalyst without NTP treatment. This confirms that oxidation is the main mechanism for coke removal by the NTP-O_2_.

We found that the NTP field strength, operation temperature, and the nature of the discharge gas were all able to influence the coke removal effectiveness; these results are presented in section Evaluation of Coke Removal by NTP. More importantly, we discovered NTP's positive role in regenerating the deactivated catalyst *in situ* for the glycerol-to-acrolein conversion (section Evaluation of *in situ* Catalyst Regeneration by NTP); a comparison of the results for the different discharge gases is also presented hereafter.

### Evaluation of Coke Removal by NTP

#### Effect of NTP Field Strength

**Figure 4:**
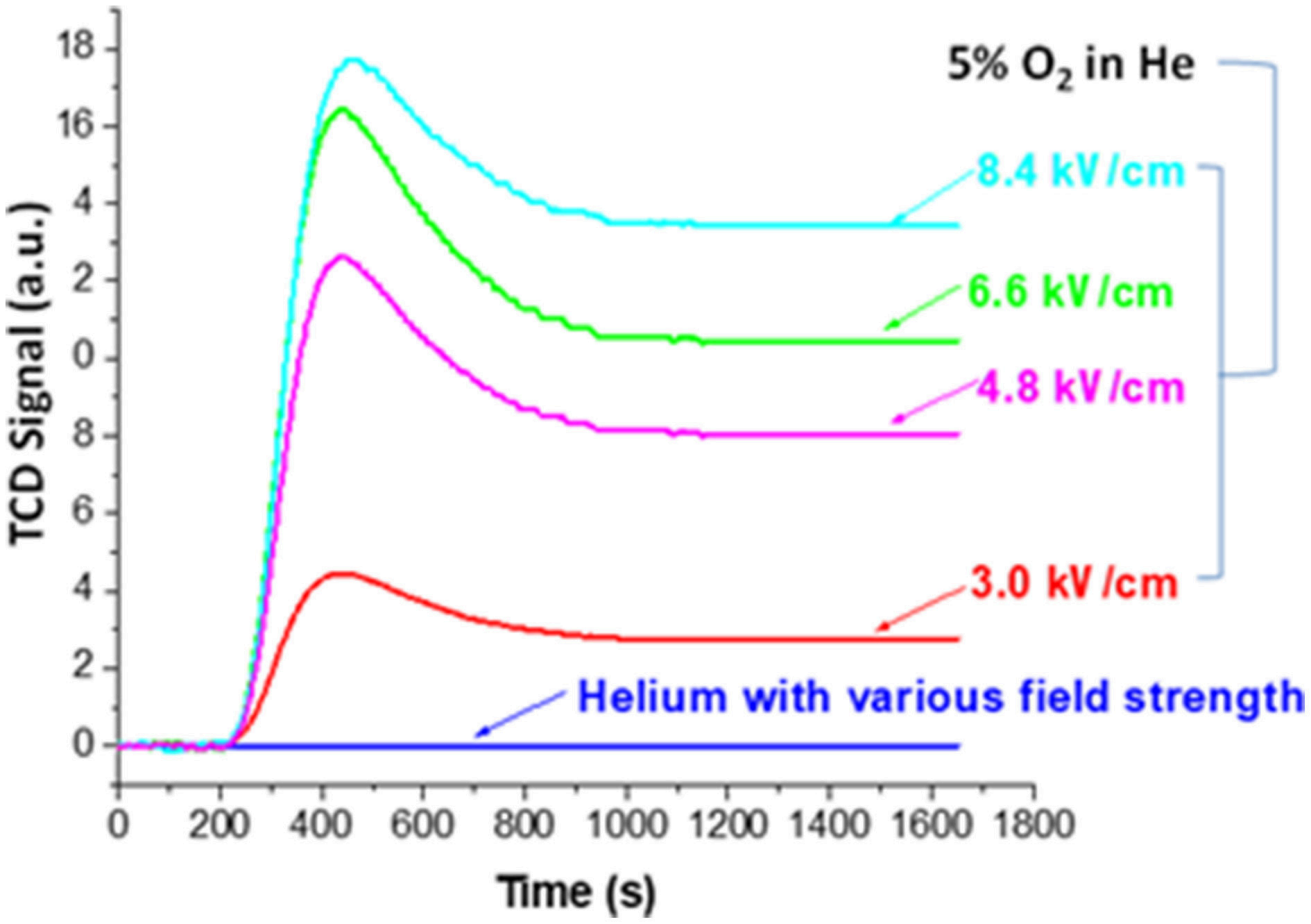
Baselines of TCD signal collected for monitoring decoking process using fresh catalyst at different field strengths with pure He or 5% O_2_ in He.

The effect of the NTP field strength was first studied by monitoring the decoking process (section Evaluation of Coke Removal by NTP Test 1). Here we first explain how the real-time TPR monitoring served the function of examining coke removal from the baseline signal. Figure 4 displays the baseline signals obtained using fresh HSiW-Si with different NTP field strengths. The baseline collected on the fresh catalyst with pure helium showed no baseline shift regardless of how large a NTP discharge field strength was applied, suggesting that the excited helium species were transient and vanished as soon as they left the discharge zone. On the contrary, the background collected on the fresh catalyst with 5% O_2_ in He mixture showed some signal changes when the NTP was applied. Each baseline curve of NTP-O_2_ displayed a characteristic initial overshoot followed by stabilization to a constant. An increasing trend of the stabilized baseline was observed as the NTP field strength increased from 3.0 to 8.4 kV/cm.

The NTP discharge excited molecular oxygen into new species, such as ozone, O (^1^D), O (^3^P) and other surface oxygen species. Except for ozone, all the other strong oxidants were short-lived. As soon as these short-lived oxygen species left the discharge zone, they recombined back to molecular oxygen. Ozone (O_3_) has a lifetime of over a day at room temperature, so it would not convert back to molecular oxygen in the effluent after leaving the discharge zone. Therefore, the gaseous effluent after the discharge cell packed with fresh catalyst was composed of O_2_, O_3_ and He. However, the O_3_ condensed and could be observed as a layer of navy-blue liquid in the liquid nitrogen trap; it could not reach the TCD detector. Therefore, essentially what TCD could detect was the consumption of oxygen. The initial overshoot was probably caused by the presence of porous materials (HSiW-Si) that adsorbed some O_2_ at different field strengths. An increasing trend of the stabilized baseline was observed as the field strength of the plasma increased from 3.0 to 8.4 kV/cm, suggesting that the ozone yield increased along with the increase of the NTP field strength. For a given condition, the ozone concentration should be a constant. Therefore, after subtracting the baseline from the signal obtained during the NTP-O_2_ treatment of the spent HSiW-Si, which was at least eight times larger than the baseline signal, any signal change would be caused by the ongoing reaction with the carbonaceous coke species deposited on the catalyst surface.

**Figure 5:**
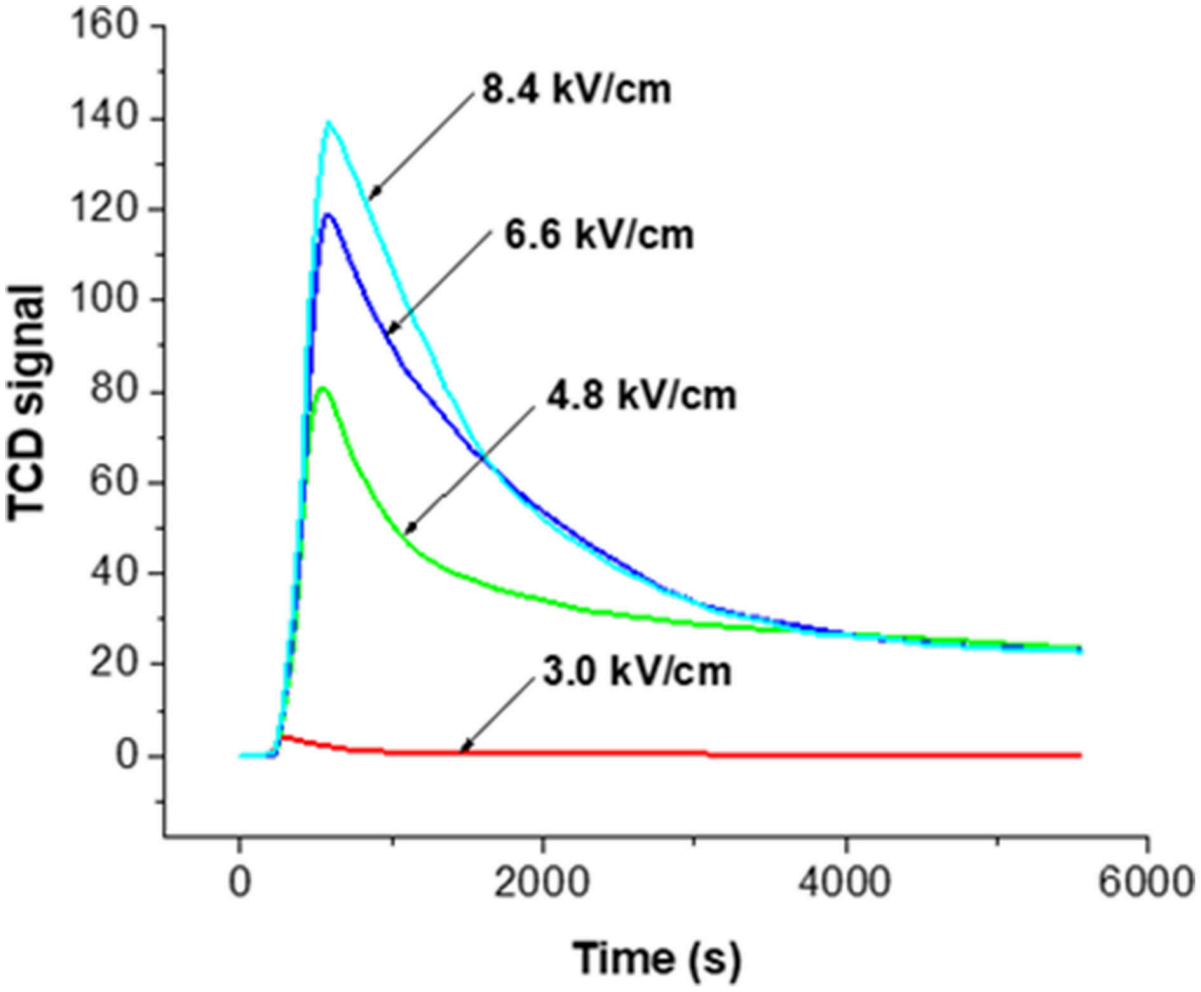
TCD signals for the real-time monitoring of the decoking process at different NTP field strengths at room temperature; 3.0, 4.8, 6.6, and 8.4 kV/cm indicate the field strength of NTP-O_2_ with 5% O_2_ in He.

Figure 5 shows the result of monitoring the decoking process at the various NTP field strengths after subtracting the corresponding baseline. There was an equilibrium between the distribution of molecular oxygen, which could be detected by TCD, and other O species generated by NTP. When the reactive O species reacted with some surface carbonaceous coke, the consumption of these species drove forward the reactions of O_2_ to the reactive species, such as ozone and O radicals, causing the consumption of O_2_. Upon each NTP application with different field strength, the signal first reached to a maximum, indicating fast oxidation of coke and consumption of O_2_. Coke was the limiting reactant while the oxidative species formed in NTP-O_2_ were in excess. It is reasonable to state that “soft” coke was consumed first, leaving the “hard” coke to be oxidized at slower rates, as indicated by the level-off of the TCD signal.

Therefore, Figure 5 implies that the effectiveness of coke removal increased with increasing NTP field strength; the effectiveness rose in the following order: 3.0 kV/cm < 4.8 kV/cm < 6.6 kV/cm < 8.4 kV/cm. It is noteworthy that at the field strength of 3.0 kV/cm, there was barely any coke removal. From 3.0 to 4.8 kV/cm, there was a drastic change in coke removal, indicating the activation of decoking by the NTP. We used a Lissajous figure to calculate the NTP power consumption, a method developed by Manley (Manley, 1943) that has been used in many studies [e.g., (Kraus et al., 2001; Subrahmanyam et al., 2005)]. The calculated power was as 0.05, 0.43, 0.84, and 1.27 W, corresponding to the field strength of 3.0, 4.8, 6.6 and 8.4 kV/cm, respectively.

**Figure 6:**
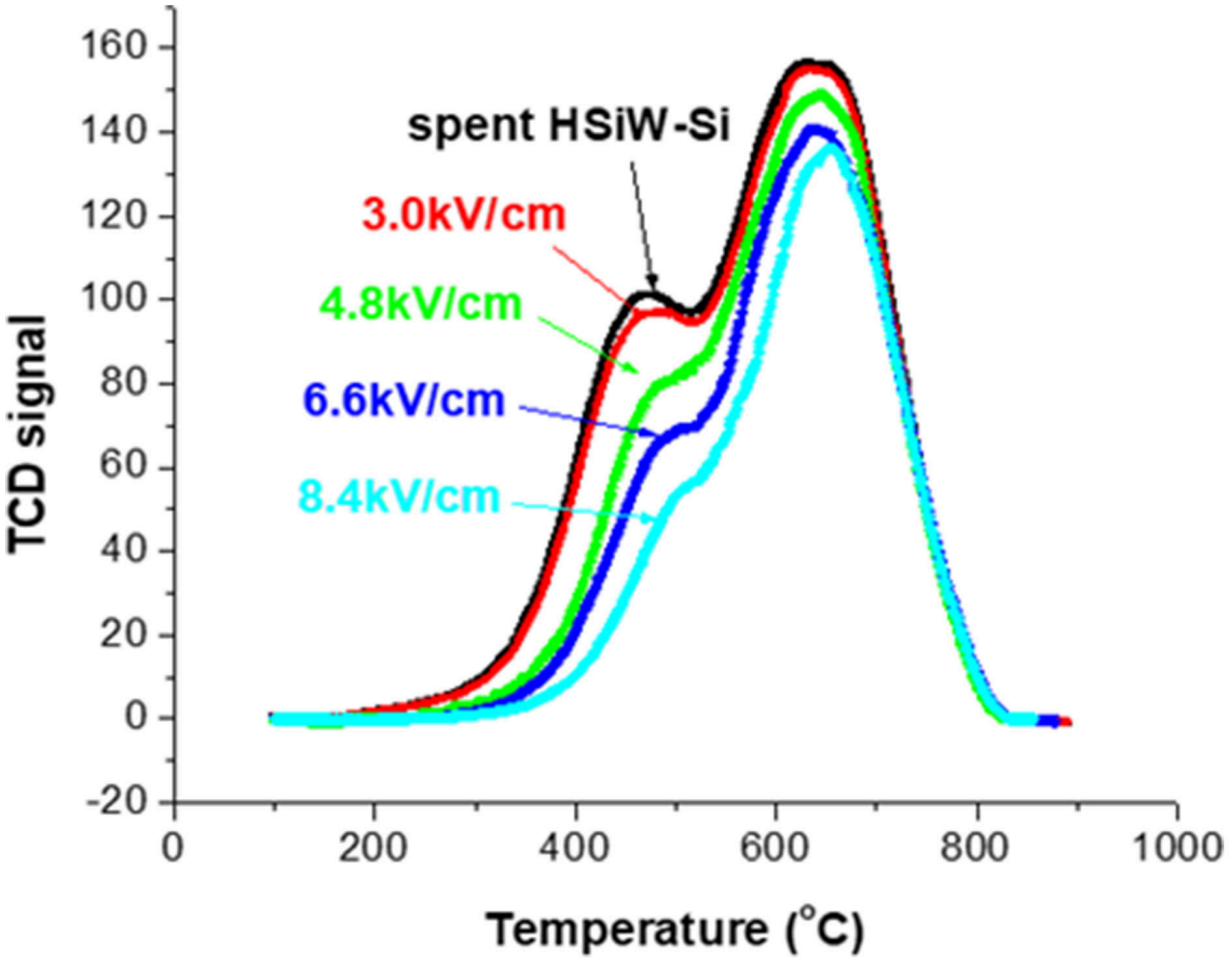
TPO characterization of the spent catalyst both without NTP-O_2_ treatment and after regeneration with NTP-O_2_ at different field strengths.

Figure 6 presents the TPO characterization (section Evaluation of Coke Removal by NTP Test 2) result of the spent catalyst without NTP treatment, and after 1.5 h of NTP-O_2_ treatment at different NTP field strengths. There is only a slight difference in the TPO profile between the untreated spent catalyst and the spent catalyst after the 1.5-h treatment by NTP-O_2_ at the field strength of 3.0 kV/cm. For the rest of the investigated field strengths, the TPO curves showed a decreasing trend with the increasing NTP field strength. Although the reduction of TCD signal intensity was more significant in the “soft” coke region (peak at ~450°C), the reduction was also observed in the “hard” coke region (peak at ~650°C).

The results of the above two tests showed that the coke removal was more effective at higher NTP field-strengths. Therefore, the field strength of 8.4 kV/cm, at which the maximum coke removal was observed, was employed for the following tests.

#### Effect of Operation Temperature

**Figure 7:**
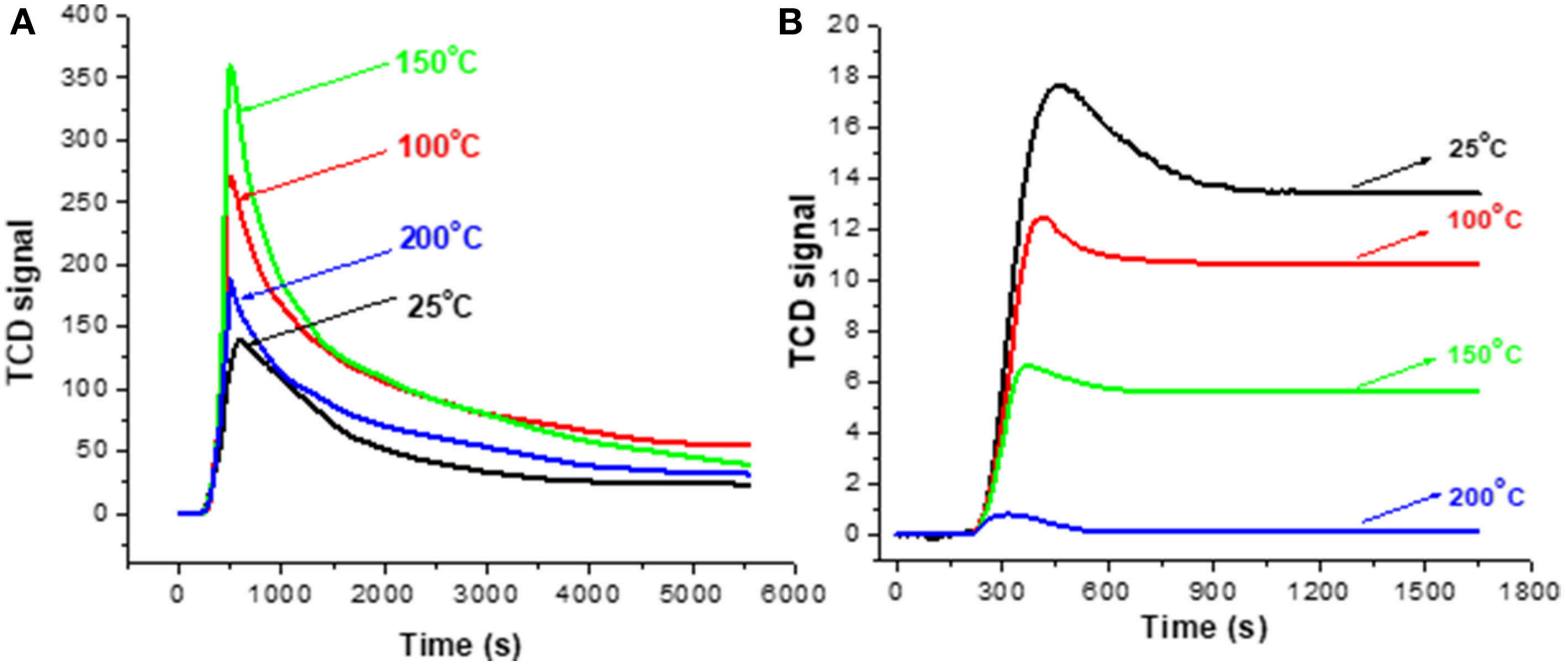
TCD signals for the real-time monitoring of the decoking process with 8.4 kV/cm NTP-O_2_ at different temperatures **(A)**; baseline profiles collected using fresh catalyst **(B)**.

Test 1 (section Evaluation of Coke Removal by NTP) was repeated with extra control of the DBD cell temperature and Figure 7 depicts the results of monitoring the decoking process. The stabilized baseline using fresh HSiW-Si (Figure 7B) decreased in the order of 25°C > 100°C > 150°C > 200°C, and the stabilized baseline at 200°C was nearly zero. The relative positions of the stabilized baselines at different operation temperatures suggest that ozone (O_3_) concentration decreased as the operation temperature increased. It is well known that ozone decomposes faster at high temperatures; almost no O_3_ existed at 200°C. Figure 7A displays the signal profile during NTP-O_2_ treatment at different temperatures. The signal intensity was descending in the order of 150°C > 100°C > 200°C > 25°C, suggesting that the effectiveness of the coke removal decreased in this order. Therefore, the order of coke removal effectiveness does not quite follow the sequence of the ozone concentration as suggested in Figure 7B. This inconsistency implies that the ozone concentration was not the major factor that determined the effectiveness of the coke removal.

**Figure 8:**
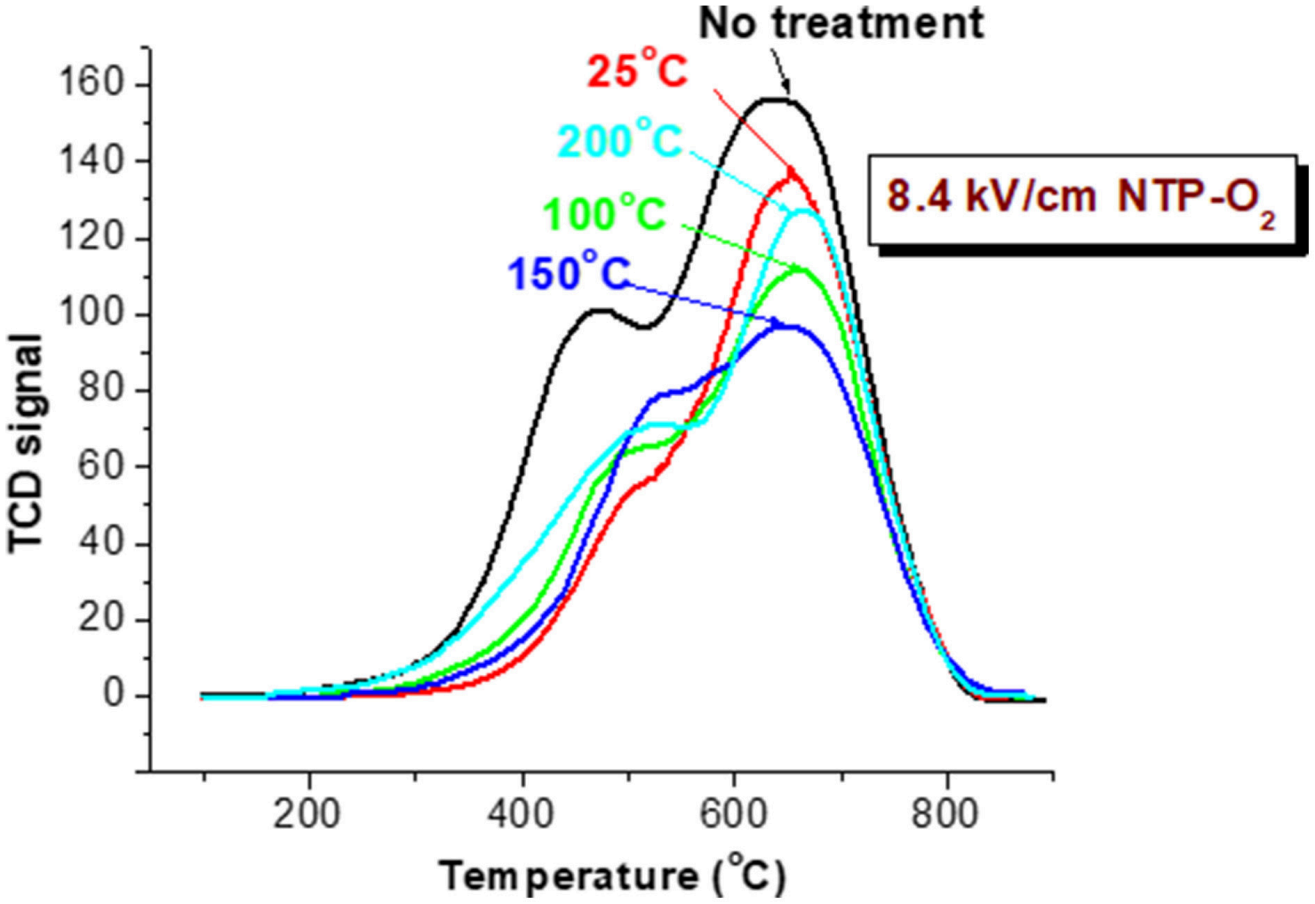
TPO characterization of spent catalyst after decoking with NTP-O_2_ of 8.4 kV/cm at different temperatures for 1.5 h.

A consistent trend was exhibited by TPO characterization of the spent HSiW-Si after the 8.4 kV/cm NTP treatment at different temperatures, as shown in Figure 8. Coke was largely reduced after the NTP-O_2_ treatment at 100°C, and even more so at 150°C. However, the trend reversed as the temperature further increased to 200°C, and more coke, especially hard coke, remained on the catalyst compared to those being treated at 100 and 150°C. The results also confirmed that the reactive oxygen species formed under NTP discharge were able to react with some hard coke (around 600–700°C), especially when the NTP regeneration was operated at a proper temperature. Among the investigated temperatures, the most effective coke removal occurred at 150°C.

Both of the TPR and TPO tests consistently showed that operating the NTP-O_2_ decoking at 150°C provided more effective coke removal compared to the other investigated temperatures.

#### Time Dependence of Coke Removal

**Figure 9:**
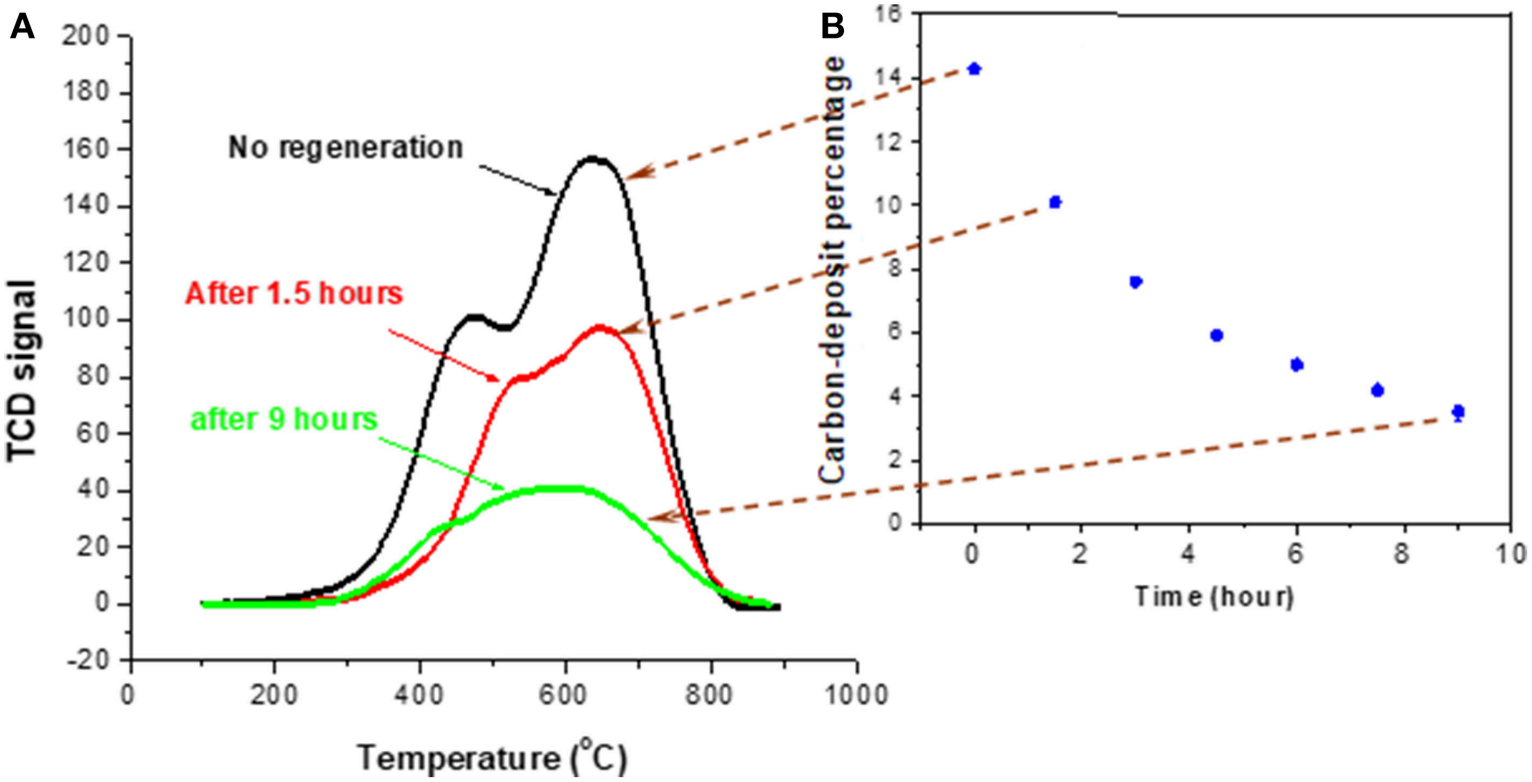
TPO characterization of spent catalyst after decoking at 150°C using 8.4 kV/cm NTP-O_2_ for different times **(A)**; percentage of coke deposit as a function of decoking time **(B)**.

Figure 9 shows the time dependence of coke removal at the NTP condition of 8.4 kV/cm and 150°C. The percentage of coke deposit was defined as the weight difference between the fresh catalyst and the coked catalyst divided by the weight of fresh catalyst. Although the strong oxidizing species in NTP were able to react with hard coke, the reaction rate is likely to be much lower in comparison to that with soft coke, which was likely removed first. This is one reason for that the decrease of coke deposit in Figure 9 became slower at the longer treatment times. Also, the diffusion of short-lived atomic oxygen into the pores was unlikely, so when the more exposed surface coke was consumed, the decoking process would become more difficult. When soft coke was oxidized, CO_2_ was formed and flushed off the surface, directly resulting in the weight decrease of the spent catalyst. In contrast, for oxidation of hard coke, the reaction rate might be slow, and the oxidation of hard coke started by converting it to an oxygen-rich intermediate, or breaking it down into a smaller structure (Deitz and Bitner, 1973; Smith and Chughtai, 1995; Mawhinney and Yates, 2001). Consequently, oxidizing hard coke most likely did not directly generate CO_2_, leaving the total mass barely changed. This conceivably explains why the coke removal rate slowed down with time, even though NTP-O_2_ was able to react with hard coke.

### Evaluation of *in situ* Catalyst Regeneration by NTP

**Figure 10:**
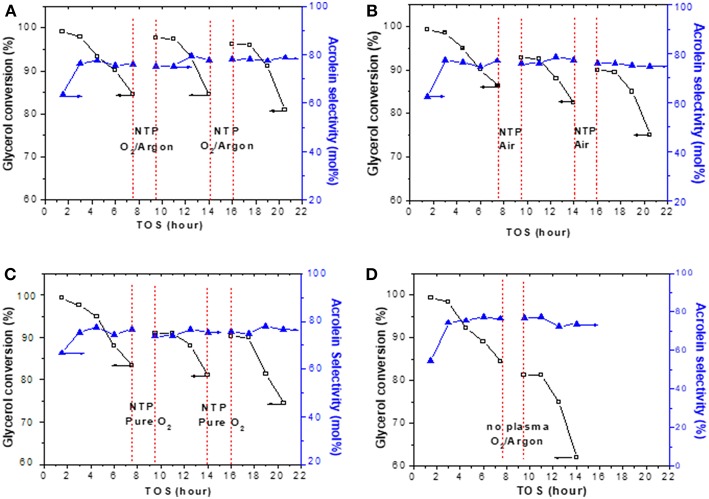
Effects of *in situ* regeneration by NTP using **(A)** 20% O_2_ in Ar, **(B)** air, and **(C)** pure oxygen, in comparison to **(D)** flushing with 20% O_2_ in Ar without NTP. Each regeneration was conducted at 8.4 kV/cm and 150°C for 2 h.

Coke removal usually resulted in catalyst regeneration, but not always, e.g., in case if some active sites on the catalyst were damaged during coke removal, catalytic performance would not be regained. The NTP-O_2_ effect in catalyst regeneration was directly tested via periodic *in situ* regeneration at 8.4 kV/cm and 150°C. The results of periodic regeneration using 20% O_2_ in argon, air, and pure oxygen are presented in Figures 10A–C, respectively. The reason that 20% O_2_ was used here was because we wanted to make the comparison of Ar as the discharge gas to N_2_ as the discharge gas (in the case of air) at similar O_2_ concentration. A control test is presented in Figure 10D, for which there was no NTP application during the 2 h of flushing the reactor with 20% O_2_ in Ar. Evidently, the NTP application regenerated the catalyst *in situ*, showing as the rebound of glycerol conversion after each session of regeneration. The NTP with 20% O_2_ in Ar showed the best effectiveness, with a descending order of effectiveness as NTP-O_2_ in Ar > NTP-pure O_2_ ≥ NTP-O_2_ in N_2_. In contrast, flushing the reactor with 20% O_2_ in Ar without NTP had no effect for regeneration. Based on the results shown previously in Figure 9, a 2-h NTP treatment was unlikely to completely remove surface coke. The results here show that the catalytic activity could be regained to some extent even with the partial regeneration. However, because of the fact that coke was not thoroughly removed, the catalyst after partial regeneration might be prone to somewhat faster deactivation. This explains why, overall, a slightly decreasing trend in glycerol conversion was observed after each regeneration. Nevertheless, the purpose of this experiment to examine the influence of the discharge gas on the regeneration was achieved.

## Discussion

### Overall Discussion

**Figure 11:**
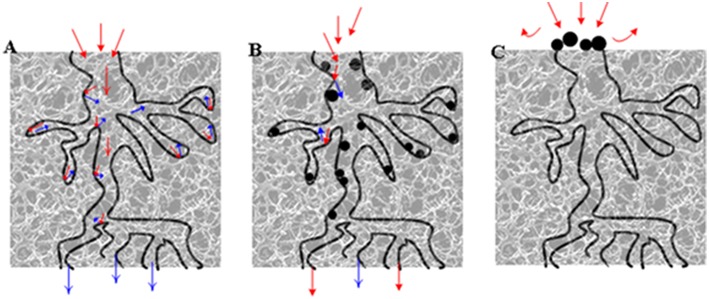
Illustration of catalyst pores **(A)** fresh catalyst; **(B)** coke relatively evenly distributed on the active sites, and glycerol molecules only could not reach the active sites covered by coke (direct deactivation); **(C)** coke deposit on the mouth of pore entrance, and glycerol molecules could not reach the active sites that were still “available” (indirect deactivation). Red arrows denote the path of glycerol, blue arrows denote the path of product (acrolein), and the black dots denote the coke.

Regarding that partial coke removal could significantly restore catalytic activity, the “indirect deactivation mechanism” (Tanabe, 1989) was probably the predominant mechanism causing the deactivation of the HSiW-Si catalyst during glycerol dehydration. Figure 11 illustrates this “indirect deactivation mechanism.” Silica had relatively small pores (average ~11 nm). Some coke formed near the mouth of the pores, narrowing or blocking the entrance into the pores, wherein lots of active sites were located (Figure 11C). All of these internal active sites became inaccessible if the pore entrance was blocked. Similarly, once these “entrance blockers” were removed or partially removed, a significant amount of active sites became accessible again for the reactant to contact. Therefore, even partial coke removal greatly revived the catalytic activity.

The situation of *in situ* NTP-O_2_ with the presence of the catalyst is complex, and to the best knowledge of the authors, such a situation has rarely been discussed in the past. In the NTP-O_2_ plasma system, the possible oxygen species include ozone (O_3_), O_2_ molecules in the ground state (O_2_(X^3^Σ^−^)), O_2_ molecules in the excited state (O_2_(a^1^Δ^−^) and O_2_(b^1^$\Sigma_{\text{g}}^{+}$)), atomic oxygen in the ground state (O(^3^P)), atomic oxygen in the excited state (O(^1^D)), and oxygen ions (${\text{O}}_{2}^{+}$, O^+^, and O^−^). Among them, the most effective oxidants are ozone and atomic oxygen. Since non-thermal plasma is an electron-driven process (Fridman, 2008), atomic oxygen is most likely to be firstly generated via dissociation in the Schumann-Runge band (Equation 3) and in the Herzberg band (Equation 4; Falkenstein, 1999). Herzberg-band dissociation has a higher efficiency than Schumann-Runge-band dissociation (Falkenstein, 1999), because the ground-state oxygen atom O(^3^P) is relatively more stable than the excited-state oxygen atom O(^1^D), and also because O(^1^D) could be easily quenched into O(^3^P) (Equation 5); O(^3^P) was possibly the most predominant atomic oxidant (Bogaerts, 2009; Mok et al., 2009).

(3) $${\text{O}}_{2}+\text{e}\to \text{O}{(}^{\text{3}}\text{P})+\text{O}{(}^{\text{1}}\text{D}),\;{\text{k}}_{1}=\text{5}.{\text{0}}^{*}{\text{10}}^{-\text{8}}\text{exp}(-\text{8}.4/{\text{T}}_{\text{e}})$$

(4) $${\text{O}}_{2}+\text{e}\to \text{O}{(}^{\text{3}}\text{P})+\text{O}{(}^{\text{3}}\text{P}),\;{\text{k}}_{2}\text{=4}.{\text{23}}^{*}{\text{10}}^{-\text{9}}\text{exp}(-\text{5}.56/{\text{T}}_{\text{e}})$$

(5) $$\text{O}{(}^{\text{1}}\text{D})+\text{M}\to \text{O}{(}^{\text{3}}\text{P})+\text{M}$$

where T_e_ is electron temperature, k is the reaction rate constant, and M is any other types of molecule.

It was proven that atomic oxygen could rapidly react with carbonaceous species (Blackwood and McTaggart, 1958; Marsh et al., 1963; Pattabiraman et al., 1990). Some researchers have claimed that the atomic oxygen is a stronger oxidant than ozone, and is capable of providing a faster rate when reacting with carbonaceous species (Smith and Chughtai, 1995; Falkenstein, 1999; Pieck et al., 2005; Khan and Al-Jalal, 2006). Some plausible overall reactions are listed in the following Equations 6 through 8 (Khan and Al-Jalal, 2006). The very detailed mechanism was not quite clear; however, it was generally thought that the O attacks the carbonaceous compound preferentially via the delocalized π bonds.

(6) $${\text{C}}_{\text{x}}{\text{H}}_{\text{y}}{\text{O}}_{\text{z}}+\text{O}+\text{e}\to {\text{C}}_{\text{x}-\text{1}}{\text{H}}_{\text{y}}{\text{O}}_{\text{z}}+\text{CO}+\text{e}$$

(7) $${\text{C}}_{\text{x}}{\text{H}}_{\text{y}}{\text{O}}_{\text{z}}+\text{O}+\text{e}\to {\text{C}}_{\text{x}-\text{1}}{\text{H}}_{\text{y}-\text{1}}{\text{O}}_{\text{z}}+\text{H}+\text{CO}+\text{e}$$

(8) $$\text{CO}+\text{O}+\text{e}\to {\text{CO}}_{2}+\text{e}$$

Ozone is another effective strong oxidant that has been previously reported as being reactive with carbonaceous species (Pieck et al., 1994; Smith and Chughtai, 1995; Mawhinney and Yates, 2001; Subrahmanyam et al., 2005). Ozone is generated via three-body collisions (Equation 9), involving the molecular oxygen, the ground-state atomic oxygen, and another gas molecule M; ozone is decomposed via Equation (10) (Mok et al., 2009). Both processes consume some atomic oxygen. The general process of ozone reacting with carbonaceous species is summarized in Equation (11) (Smith and Chughtai, 1995).

(9) $${\text{O}}_{2}+\text{O }{(}^{\text{3}}\text{P})+\text{M}\to {\text{O}}_{3}+\text{M},\;{\text{k}}_{3}=\text{8}.{\text{6}}^{*}{\text{10}}^{-\text{31}}{\text{T}}^{-\text{1}.\text{25}}$$

(10) $${\text{O}}_{3}+\text{O }{(}^{\text{3}}\text{P})\to 2{\text{O}}_{2},\;{\text{k}}_{2}=\text{9}.{\text{5}}^{*}{\text{10}}^{-\text{12}}\text{exp}(-\text{2300/T})$$

where T is the overall gas temperature.

(11) $$\begin{array}{l} {\text{C}}_{\text{n}}+{\text{O}}_{3}\to \text{Functional groups }(-\text{COOH},-\text{C}-\text{OH},\;-\text{C}\\ \;=\text{O})\;\to \ldots \to {\text{CO}}_{2} \end{array}$$

Possible mechanisms of ozone reaction with the functional groups in surface coke have been discussed previously (Deitz and Bitner, 1973; Smith and Chughtai, 1995; Mawhinney and Yates, 2001). For examples, if a carbon-carbon double bond was located at the end of an aliphatic carbon chain, the ozone addition could directly reduce the number of carbons and release one mole of CO_2_; if a carbon-carbon double bond was located within an aromatic carbonaceous compound, then the ozone insertion may have opened up the ring structure, significantly lowering its stability and thus lowering the activation energy for further oxidization and increasing the reactivity. The process could have continued in a step-by-step fashion, breaking the larger molecule into smaller molecules, and eventually converting all to CO_x_, which could be pumped out of the system.

Some of the oxidizing species could also chemically bind to the catalyst surface, existing as oxidizing surface-species. The active surface species are assigned to three categories: surface atomic oxygen (denoted as O-S), adsorbed excited oxygen (${\text{O}}_{2}^{*}$-S) and adsorbed ozone (O_3_-S) (Equations 12–14). It is possible that the reactions with the carbonaceous species proceeded via two different pathways. First, the active O species in the gas phase directly attacked the surface coke, which would be O_3_, O, or O^*^ directly interacting with coke. Second, the reactive surface O species (O-S, ${\text{O}}_{2}^{*}$-S, and O_3_-S) migrated to the surface and reacted with the neighboring coke.

(12) $${\text{O}}_{3}+\text{S}\to {\text{O}}_{3}-\text{S}$$

(13) $${\text{O}}_{2}{(}^{\text{1}}\Delta \text{P})+\text{S}\to {\text{O}}_{2}{(}^{\text{1}}\Delta \text{P})-\text{S }({\text{O}}_{2}^{{\;}^{*}}-\text{S})$$

(14) $$\text{O}{(}^{\text{3}}\text{P})+\text{S}\to \text{O}{(}^{\text{3}}\text{P})-\text{S or O}{(}^{\text{1}}\text{D})+\text{S}\to \text{O}{(}^{\text{1}}\text{D})-\text{S}$$

Conventional combustion method of a substance with molecular oxygen is an exothermal process. The operation requires a very strict temperature controlling and heat release system to avoid temperature runoff, which would result in the permanent deactivation of catalyst (such as sintering; Pieck et al., 1994), and more importantly, potential safety issues for a plant. Regeneration with NTP-O_2_ at a mild temperature condition could best preserve the catalyst properties. Therefore, in general, it would be beneficial if a process that can remove coke at a low temperature is developed. The coke on the acid catalyst HSiW-Si from glycerol dehydration was more distributed toward the hard coke region compared to most spent cracking metal catalysts (Tanable et al., 1989; Li and Brown, 1999). Therefore, our concept proved for a more difficult model compound (coked acid catalyst HSiW-Si) is very likely to be applicable to metal catalysts, which are sensitive to sintering. Some modifications of the catalyst, such as doping with palladium metals (Kozhevnikov, 2007), may significantly modify the coke distribution (to the softer end) and lead to more efficient regeneration.

### Effects of NTP Field Strength (Figures 5, 6)

As the field strength increased, the electrons became more energetic and a larger number of energized electrons was generated in the system. As a result, the dissociation of the molecular oxygen was facilitated, and more atomic oxygen was formed. Ozone was generated via the three-body collision, and atomic oxygen was the limiting substrate. Therefore, if all other parameters remained the same, the ozone concentration was likely to increase as the field strength increased (Teranishi et al., 2009). With the application of a more intense plasma field, more reactive oxygen species were generated, and more energy that could be efficiently transferred to the oxidation processes was available in the system. As a result, coke removal effectiveness increased along with the field strength. However, there were also decomposition/recombination reactions that converted the reactive oxygen species back to molecular O_2_, as shown in Equations 15 and 16. As a result, the density (or concentration) of ozone and atomic oxygen would not further increase as the NTP field strength increased, and it was more likely to be an asymptotic function of field strength increasing toward a saturation point. This statement agrees with a previous finding by Sung et al. that ozone concentration in an atmosphere of oxygen increased with the increase of the discharge power until approaching a saturation point (Sung and Sakoda, 2005). This explained that while coke removal increased with NTP field strength, the increasing trend leveled off.

(15) $${\text{O}}_{3}+\text{O }{(}^{\text{3}}\text{P})\to \text{2 }{\text{O}}_{2}$$

(16) $$\text{O }{(}^{\text{3}}\text{P})+\text{O }{(}^{\text{3}}\text{P})(\text{+M})\to {\text{O}}_{2}+\text{e }(\text{+M})$$

### Effects of Operation Temperature (Figures 7, 8)

The experimental results showed that operating the NTP-O_2_ regeneration at 150°C provided more effective coke removal than operating at any other temperatures investigated. The majority of oxidizing species were ozone and atomic oxygen, and maybe some OH radicals. The kinetic equations for ozone show that low temperature favors ozone formation (Equation 9), while high temperature favors ozone decomposition (Equation 10); both processes consume some atomic oxygen. At higher temperatures, less ozone is formed. Because the ozone concentration is reduced in the first place, there is a limited amount of atomic oxygen that would be consequently consumed according to Equation (10). Although few ozone molecules could exist at temperature conditions above 200°C (as indicated in Figure 7B), atomization of oxygen molecules is feasible at 300°C (Khan and Al-Jalal, 2004). Some believe that atomic oxygen is an even stronger oxidizing species than ozone (Smith and Chughtai, 1995; Falkenstein, 1999; Silva et al., 2004; Pieck et al., 2005; Khan and Al-Jalal, 2006). Atomic oxygen is a short-lived species, and it may not be able to diffuse deeply into a pore within its lifetime. However, higher temperature increases the kinetic velocity of atomic oxygen, facilitating deeper penetration into the catalyst and increasing the collision probability with more carbonaceous molecules (Khan and Al-Jalal, 2006). Consequently, the reaction of atomic oxygen with coke would be enhanced. Nonetheless, when the oxygen atoms move faster at elevated temperatures, the odds of their collision with another oxygen atom increased, increasing the possibility of recombination back to molecular oxygen (Equation 17). As the temperature increases, the rate of oxidation with carbonaceous coke by atomic oxygen and/or ozone also increases significantly. All these factors interacting and counteracting with each other resulted in the optimal operation occurring at a mildly elevated temperature, thus in our study, coke removal at 150°C showed the greatest effectiveness among all the investigated temperatures.

(17) $$\text{O }{(}^{\text{3}}\text{P})+\text{O }{(}^{\text{3}}\text{P})\to {\text{O}}_{2}$$

It is possible that the fluidized bed reactor would facilitate the catalyst regeneration process, because of the complex balance existing in diffusion and lifetime for atomic oxygen and ozone.

### Time Dependence (Figure 9)

It is rather difficult for the short-lived NTP species (O(^3^P), O(^1^D) and OH) generated in the gas-phase and/or on catalyst surface to diffuse deeply into the catalyst pore due to their short lifetimes. The relatively long lifetime of ozone would make it diffuse into the pores easily (Holzer et al., 2002); however, ozone is much larger in molecular size than atomic oxygen, and its steric hindrance is therefore larger than that of atomic oxygen. Therefore, ozone would not diffuse into pores as easily as atomic oxygen because of the steric effect (Deitz and Bitner, 1973). As the result, the diffusion of highly reactive oxidants into the porous catalyst might be another important issue limiting the rate of further coke removal, slowing down the removal as the coke on top of the surface is consumed. These reasons may explain why the coke removal rate slowed down with time, even though NTP-O_2_ was able to react with hard coke. The coke removal rate decreased with the increasing regeneration time. These reactions would continue as long as the basic graphitic structure and carbon-carbon double bonds still exist (Takeuchi and Itoh, 1993), although the reaction rate varied.

### Effects of Discharge Gas (Figure 10)

Plausible reactions under NTP with different background gases are listed in Table 1 (Khan and Al-Jalal, 2004; Sung and Sakoda, 2005). The metastable state of argon (Ar^M^), formed from Ar radical (Ar^*^) via radioactive decay, had a very small probability of transition for further decay, and instead, they were more likely to transfer energy to oxygen molecules via collisions, generating O atoms and thus ozone. Also, the presence of a large number of Ar atoms created an inhibiting “wall” between oxygen atoms and oxygen atoms or ozone, preventing these active oxygen species from recombining into molecular oxygen. Consequently, the density of atomic oxygen and ozone in the presence of Ar as the background gas might be higher than that when pure O_2_ was used. Similarly, the background N_2_ could also have provided the same “wall-effect.” However, ionizing N_2_ requires much higher energy/field strength, and the ionized N species might react with the active oxygen species, forming NO_x_ (Fridman, 2008); such reactions competitively consumed a part of the highly reactive oxygen species, resulting in a decrease in the regeneration effectiveness. Generally, a noble gas, such as Ar and He, can be ionized most easily. Therefore, at a given NTP field strength, the electron energy and the number of electrons were significantly higher in the system with Ar as a background gas than those in the other two systems (Snyder and Anderson, 1998; Okumoto et al., 2001). Forming atomic N, comparing to forming atomic Ar or O, costs a much larger amount of energy (Fridman, 2008), and the dissociation of nitrogen competitively consumed a certain amount of energy available in the system. The presence of nitrogen added to the complexity of the gaseous-electron processes and downgraded the electron energy density. Studies have shown that the rate of atomic oxygen formation in a mixture with argon was an order of magnitude higher than that in a mixture with nitrogen (Snyder and Anderson, 1998; Khan and Al-Jalal, 2006). These factors might account for our result in that the regeneration effectiveness decreased as NTP-O_2_ in Ar > NTP-O_2_ > NTP-O_2_ in N_2_. All the discussion associated with argon should be presumably applicable to any noble gas, such as helium.

**Table 1:** Relevant reactions in NTP-O_2_ conditioned with different discharge gases.

**Discharge gas^a^**	**Relevant reactions in the specific NTP-O_**2**_**
O_2_	e + O_2_ → 2 O + e e + O_2_ → ${\text{O}}_{2}^{+}$ + 2e e + ${\text{O}}_{2}^{+}$ → 2 O ^*^
Ar	e + Ar → Ar^*^ + e where Ar^*^ is the excited state, or Ar radical
	Ar^*^ → Ar^M^ where Ar^M^ is the metastable states of Ar from Ar
	Ar^M^ + O_2_ → Ar + O + O Ar^M^ + O → Ar + O^*^
	Ar^+^ + O_2_ → ${\text{O}}_{2}^{+}$ + Ar ${\text{O}}_{2}^{+}$ + e → O + O
N_2_	e + N_2_ → 2 N + e e + N_2_ → ${\text{N}}_{2}^{+}$ + 2e
	O^+^ + N_2_ → NO^+^ + N ${\text{NO}}_{2}^{+}$ + N_2_ → NO^+^ + NO
	${\text{N}}_{2}^{+}$ + O_2_ → NO^+^ + NO

a *The gas component present with a larger percentage than O_2_; to be specific, O_2_ relates to our case of using pure oxygen; Ar relates to our case of using 20% O_2_ blended in argon; N_2_ relates to our case of using air (~20% O_2_ blended in N_2_)*.

## Conclusions

Silica-supported silicotungstic acid (HSiW-Si), which shows good performance in glycerol dehydration initially but deactivates quickly, was chosen to probe the potential of using non-thermal plasma with oxygen-containing gas (NTP-O_2_) to solve the catalyst deactivation problem. This study proved that NTP-O_2_ is a viable method to regenerate supported HSiW, which cannot be accomplished by the conventional combustion method due to its low thermal stability. The existence of strong oxidizing agents produced in NTP (e.g., O_3_, atomic oxygen, and active surface oxygen species) made it possible to oxidize coke at a much lower temperature compared to the conventional combustion method. NTP-O_2_ at higher field strengths was more effective in coke removal; however, the improvement in the coke removal exhibited an asymptotic tendency, indicating the limitation or slow rate in removing hard coke. Among the investigated field strengths and temperature conditions, operating NTP-O_2_ at 8.4 kV/cm and 150°C provided the most effective coke removal. Longer NTP-O_2_ treatment time to the spent catalyst would certainly regenerate the catalyst more thoroughly; however, a balance between the treatment time and the regeneration outcome needs to be found, since the coke removal becomes slower while longer time means more energy consumption.

It is likely that HSiW-Si was deactivated via an indirect mechanism, since partial decoking by NTP-O_2_ could regenerate the catalyst to a large degree. NTP-O_2_ with argon as background gas showed better regeneration effectiveness than that with nitrogen as background gas; also, diluting oxygen with argon showed better regeneration effectiveness than using pure oxygen. This comparison is presumably applicable to other noble gases like helium as well.

NTP-O_2_ could react with both soft coke and hard coke. For the soft coke, NTP-O_2_ could likely convert it to CO/CO_2_ that could be flushed off catalyst surface, resulting in weight loss of the spent catalyst. On the other hand for the hard coke, the active oxidants in NTP reacted with them and formed some intermediate oxygenated surface compounds, shifting the coke distribution to the soft coke. NTP-O_2_ could significantly lower coke-removal temperature, and this method is presumably applicable to other catalysts (and other reactions) with low thermal stability that suffer from coking deactivation.

## Author Contributions

XY conceived the study and directed the experimental work conducted by LL. Catalyst characterizations were done collaboratively by LL, XY, BK, MC, SP, and FD. LL drafted the manuscript. All authors participated in the revision and approved the manuscript.

### Conflict of Interest Statement

The authors declare that the research was conducted in the absence of any commercial or financial relationships that could be construed as a potential conflict of interest.
