# Supplementary material for: Extending Catalyst Life in Glycerol-to-Acrolein Conversion Using Non-thermal Plasma
Source: Front Chem. 2019 Mar 1;7:108. doi: 10.3389/fchem.2019.00108 (PMC6405481; doi:10.3389/fchem.2019.00108)
Supplement: Supplementary file 1 [file Data_Sheet_1.docx]

Supplementary Material

**Extending Catalyst Life in Glycerol-to-acrolein Conversion Using Nonthermal Plasma**

Lu Liu^1^, X. Philip Ye^1^*, Benjamin Katryniok^2^, Mickaël Capron^2^, Sébastien Paul^2^, Franck Dumeignil^2^

^1^ Department of Biosystems Engineering and Soil Science, The University of Tennessee, Knoxville, TN, United States

^2^ Univ. Lille, CNRS, Centrale Lille, ENSCL, Univ. Artois, UMR 8181 – UCCS – Unité de Catalyse et Chimie du Solide, Lille, France

* Correspondence author:

Dr. X. Philip Ye: [xye2@utk.edu](mailto:xye2@utk.edu)

Powder X-ray diffraction (XRD) patterns were recorded on a Philips X’Pert PRO PW3050 X-ray diffractometer using Cu-*Kα* radiation (0.154 nm) and a graphite generator. The diffractometer was operated at an accelerating voltage of 45 kV, scanning from 2° to 80° 2θ range with a scan rate of 0.5°/min.

Supplementary Figure S1: XRD patterns of fresh HSiW-Si and spent HSiW-Si.

Solid-State Cross-Polarization Magic Angle Spinning Carbon-13 [Nuclear Magnetic Resonance](https://www.sciencedirect.com/topics/chemistry/nuclear-magnetic-resonance) (CP/MAS ^13^C-NMR) analysis was performed on a Bruker Avance 400 MHz (9.4 T) spectrometer operating at Larmor frequency equal to 100 MHz for ^13^C. A zirconia rotor (4 mm) spinning at a MAS frequency of νMAS = 10 kHz was used. ^13^C chemical shift was referenced relative to tetramethylsilane (TMS, *δ* = 0 ppm). Recycle delay for all the CP experiments was 3 s, and TPPM decoupling was applied during signal acquisition. Cross-polarization transfers were performed using adiabatic tangential ramps to enhance the transfer efficiency, and the CP contact time was equal to 3 ms. The spectra have been decomposed with DMFIT (2004) software.

Supplementary Figure S2: CP/MAS ^13^C-NMR spectrum of spent HSiW-Si without NTP-O_2_ treatment versus that treated with NTP-O_2_.
